# Preparation and Properties of Thermoregulated Seaweed Fibers Based on Magnetic Paraffin wax@calcium Carbonate Microcapsules

**DOI:** 10.3390/ma17194826

**Published:** 2024-09-30

**Authors:** Yonggui Li, Congzhu Xu, Yuanxin Lin, Xiaolei Song, Runjun Sun, Qiang Wang, Xinqun Feng

**Affiliations:** 1 Faculty of Clothing and Design, Minjiang University, Fuzhou 350108, China; 15750843517@163.com (Y.L.); sxlyanc@126.com (X.S.); w_q2000@aliyun.com (Q.W.); 2 Fujian Key Laboratory of Novel Functional Textile Fibers and Materials, Minjiang University, Fuzhou 350108, China; xu15005932521@163.com; 3 College of Textile Science and Engineering, Xi’an Polytechnic University, Xi’an 710048, China; sunrunjun@xpu.edu.cn; 4 College of Fashion and Design, Donghua University, Shanghai 201620, China; fly6816_lele@163.com

**Keywords:** magnetic paraffin wax@calcium carbonate microcapsules, phase change materials, wet spinning, thermoregulation, seaweed fibers

## Abstract

In order to enhance the application of thermoregulated materials, magnetic phase change microcapsules were prepared using a self-assembly method. Paraffin wax was chosen for its fine thermoregulation properties as the core material, while Fe_3_O_4_ nanoparticles doped in calcium carbonate served as the hybrid shell material. The microcapsules were then blended with sodium alginate and processed into seaweed fibers through wet spinning. The microstructure, thermal, and magnetic properties of the microcapsules were analyzed using scanning electron microscopy, energy dispersive X-ray spectroscopy, a laser particle size analyzer, an X-ray diffractometer, a differential scanning calorimeter, a thermogravimetric analyzer, and a vibrating sample magnetometer. The thermoregulation of the fibers was evaluated using a thermal infrared imager. The results indicated that the microcapsules had a uniform size distribution and good thermal properties. When the mass fraction of Fe_3_O_4_ nanoparticles was 8%, the microcapsules exhibited a saturation magnetization of 2.44 emu/g and an enthalpy value of 94.25 J/g, indicating effective phase change and magnetic properties. Furthermore, the thermoregulated seaweed fibers showed a high enthalpy value of 19.8 J/g with fine shape, offering potential for developing multifunctional fiber products.

## 1. Introduction

As the continuous expansion of the global economy and industrialization progresses, human demand for energy is on the rise. Concurrently, the challenges of energy shortages and environmental pollution have become significant global concerns, attracting widespread social attention [1] To address these issues, the development of efficient energy utilization technologies has become crucial. Among various technologies, thermal energy storage (TES) is distinguished by its unique capability to balance the supply and demand of thermal energy and effectively distribute energy across time and space [2,3] Phase change materials (PCMs) are essential for the progress of thermal energy storage (TES) technology. They show significant promise in fields such as textiles, medicine, smart homes, military applications, and architecture by efficiently storing and releasing thermal energy through phase transitions at specific temperatures [4]. In the textile industry, PCMs can effectively regulate the microclimate around the wearer, ensuring comfort even in extreme environments. By storing or releasing latent heat within a designated temperature range, these thermoregulated textiles enhance quality of life and pave the way for innovative textile products [5].

Paraffin wax (PW), known for its excellent latent heat storage capacity, chemical stability, and significant phase change heat within a specific temperature range [6,7], has been extensively researched as a quintessential phase change material. Despite these benefits, practical applications reveal challenges such as low thermal conductivity and potential leakage during phase transitions. To address these issues, coating technology is often employed. Organic coatings like urea-formaldehyde resins, dense amine resins, and polymethylmethacrylate are commonly used to create phase transition microcapsules. However, these materials exhibit drawbacks, including insufficient mechanical strength, vulnerability to aging, and poor thermal conductivity, motivating researchers to seek more robust alternatives [8,9]. Utilizing inorganic materials or incorporating highly thermally conductive nanoparticles as coatings has proven effective [10]. Among inorganic materials, calcium carbonate (CaCO_3_) stands out for its high thermal conductivity, wide availability, and cost-effectiveness. Using CaCO_3_ as a coating for phase change microcapsules can effectively enhance their thermal conductivity and mechanical properties, overcoming the limitations of organic coatings [11,12]. Wei et al. [13] utilized self-assembly technology to successfully synthesize microcapsules with paraffin cores and Ce^3+^-doped CaCO_3_ shells. Their findings indicate that incorporating rare earth elements not only yielded a more uniform spherical structure but also enhanced the thermal regulation capabilities and packaging efficiency of the microcapsules. This innovative method opens new prospects for the development of multifunctional microcapsules in research. Similarly, Jiang et al. [14] prepared environmentally friendly phase change microcapsules using paraffin as the core material and graphene-modified CaCO_3_ as the shell material. Their research demonstrates that the addition of graphene enhances the heat storage, thermal stability, and thermal conductivity of the microcapsules. Notably, when the mass fraction of graphene reaches 1%, the encapsulation efficiency of the microcapsules reaches 73.19%, highlighting its potential for broad applications in the green energy sector.

Previous studies primarily used organic materials for microcapsule shells in thermoregulated seaweed fibers. However, inorganic materials offer superior strength, fire resistance, thermal conductivity, and stability. This study is unique in two aspects: Firstly, it uses organic PW as the PCMs and CaCO_3_- doped magnetic Fe_3_O_4_ nanoparticles as the shell material, enhancing the microcapsules’ surface morphology, weak magnetism, and heat storage capacity. Secondly, biocompatible SA was combined with these magnetic microcapsules, and a wet spinning process was used to create seaweed fibers that store and release energy in response to temperature changes. These fibers are environmentally friendly and multifunctional, with promising applications in various fields.

## 2. Materials and Methods

### 2.1. Materials

In this experiment, paraffin wax (PW, melting point: 28 °C) was purchased from Dongguan Donglin New Materials Co., Ltd. (Dongguan, China). Anhydrous calcium chloride (CaCl_2_), sodium dodecylbenzene sulfonate (SDBS), Fe_3_O_4_ nanoparticles, sodium alginate (SA), and polyvinyl alcohol (PVA) were obtained from Shanghai Macklin Biochemical Technology Co., Ltd. (Shanghai, China). Sodium carbonate (Na_2_CO_3_) and anhydrous ethanol were acquired from Xilong Scientific Co., Ltd. (Chengdu, China). Deionized water was used during the experiments.

### 2.2. Preparation of Magnetic PW@CaCO_3_ Phase Change Microcapsules with Different Fe_3_O_4_ Mass Fractions

Firstly, 1.5 g of the surfactant SDBS was dissolved in 100 mL of deionized water and magnetically stirred for 20 min to ensure a homogeneous dispersion. Concurrently, 10 g of PW was melted in a three-necked flask at 40 °C using a water bath. The SDBS solution was then introduced into the flask, and the mixture was mechanically stirred at 1000 rpm for 30 min to achieve a uniform oil phase solution.

In the subsequent step, 10 g of CaCl_2_ was dissolved in 100 mL of deionized water and stirred magnetically for 20 min. Simultaneously, 9.6 g of Na_2_CO_3_ and nanoparticles of Fe_3_O_4_ with mass fractions of 3% and 8% were dissolved in 100 mL of deionized water and ultrasonically dispersed for 3 h to ensure complete dissolution and dispersion.

The CaCl_2_ solution was then added dropwise to the oil phase solution while maintaining mechanical stirring at 1000 rpm for 3 h. The endpoint of this reaction was determined by monitoring the stabilization of the solution’s viscosity. Once the viscosity stabilized, it indicated the completion of the initial reaction phase.

Subsequently, the CaCO_3_ solution containing Fe_3_O_4_ nanoparticles was added dropwise to the reaction mixture, and the stirring speed was adjusted to 800 rpm for an additional 3 h. The reaction endpoint was identified by the uniform appearance of the suspension, which ensured that the microcapsules had formed consistently.

Finally, the reaction product was washed with deionized water to remove any residual reactants and unreacted materials, ensuring the purity of the microcapsules.

### 2.3. Preparation of Thermoreguhlated Seaweed Fiber

Firstly, a certain amount of SA powder was added to deionized water, swollen in a 50 °C water bath for 2 h, and then mechanically stirred for 3 h to obtain a mass fraction of 4% SA solution. Meanwhile, a 4% CaCl_2_ solution was prepared as the coagulation bath.

Secondly, a PVA solution with a mass fraction of 2% was added to the SA solution along with PW@CaCO_3_@8% Fe_3_O_4_ phase change microcapsules. The mixture was stirred for 2 h to achieve a uniformly mixed spinning stock solution. Subsequently, the spinning solution was carefully poured into the dissolving kettle to remove any bubbles and then left undisturbed for 24 h.

Finally, the flow rate was set at 9 mL/h and the coagulation bath temperature was set at 40 °C. Seaweed fibers were prepared using the wet spinning device (TS-01-20, Changzhou, China) for further study.

### 2.4. Characterization and Measurement

The surface morphology of the microcapsules and seaweed fibers was observed using scanning electron microscopy (SEM) (Hitachi SU8010, Tokyo, Japan). The SEM detector used a 10 kV accelerating voltage for secondary electron detection. Elemental analysis of the microcapsules was performed using Energy Dispersive X-ray Spectroscopy (EDS). The particle size distribution of the microcapsules was tested using a Laser Particle Size Analyzer (LPS) (LS-POP (9), Zhuhai, China). The test was conducted after stirring and ultrasonic homogenization. The crystal structure of CaCO_3_, Fe_3_O_4_, and magnetic phase change microcapsules were analyzed using an X-ray diffractometer (XRD) (Bruker D8 Advance, Karlsruhe, Germany), CuKα lamp, wavelength λ = 1.542, scanning range from 10° to 80°, voltage 40 kV, current 40 mA. The phase change behavior of PW, PW@CaCO_3_ phase change microcapsules, magnetic PW@CaCO_3_ phase change microcapsules, and seaweed fibers were determined using a differential scanning calorimeter (DSC) (214 Polyma, Free State of Bavaria, Germany). The temperature range of the DSC measurements was set from 0 °C to 50 °C at a heating rate of 10 °C/min under a nitrogen atmosphere. The thermal stability of the process samples was tested using a thermogravimetric analyzer (TG) (209 F3, Free State of Bavaria, Germany), while the sample was heated from room temperature to 400 °C in an aluminum crucible at a heating rate of 10 °C/min. The magnetic properties of magnetic PW@CaCO_3_ phase change microcapsules were characterized using a vibrating sample magnetometer (VSM) (LakeShore 7404, Columbus, USA) from −20 KOe to 20 KOe at room temperature. The thermoregulated operation of seaweed fibers was carried out by a flat temperature retaining instrument (YG-B-606D, Wenzhou, China), and the data were recorded using an infrared thermographic camera (UTi 260A, Dongguan, China) to obtain heating and cooling time-temperature curves.

## 3. Results

### 3.1. Preparation Mechanism of Thermoregulated Seaweed Fibers

**Figure 1:**
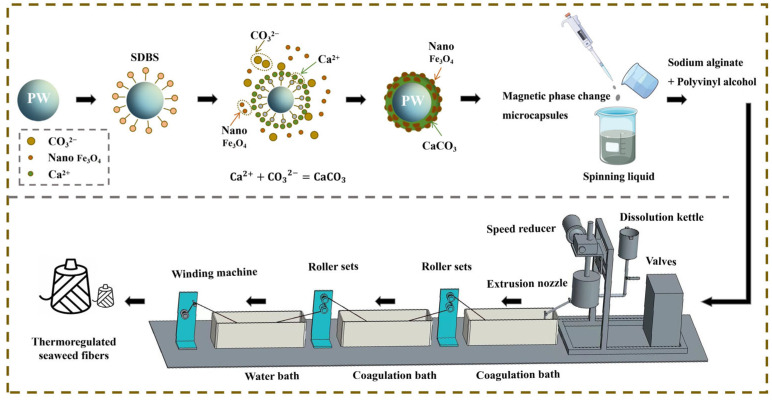
Mechanism of preparation of thermoregulated seaweed fibers.

Figure 1 illustrates the mechanism diagram of thermoregulated seaweed fibers preparation. The reaction principle of magnetic phase change microcapsules is as follows. Firstly, PW is dispersed into homogeneous particles under the action of the surfactant SDBS. At this time, the oleophilic groups of SDBS reach out to the PW particles, while hydrophilic groups reach out to the aqueous solution. Secondly, CaCl_2_ solution is added drop by drop, while Ca^2+^ approaches the PW particles due to the electrostatic effect and reacts with the hydrophilic groups to form a complex. Thirdly, Na_2_CO_3_ solution with Fe_3_O_4_ nanoparticles is continuously dripped in. Ca^2+^ and CO_3_^2−^ react to uniformly encapsulate the PW particles for hybrid shell materials. Finally, the magnetic phase change microcapsule was obtained, as shown in Figure 1. Compared to the in-situ polymerization method used by Ke et al. [15], this self-assembly method for preparing phase change microcapsules has milder reaction conditions, requiring neither high temperatures nor high pressures, thus saving energy consumption and reducing costs.

### 3.2. Surface Morphology Analysis of Microcapsules

**Figure 2:**
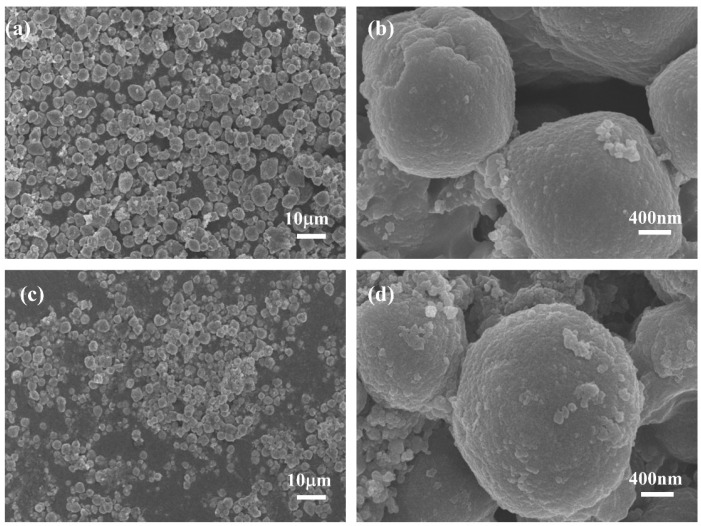
(**a**,**b**) SEM images of PW@CaCO_3_ phase change microcapsules; (**c**,**d**) SEM image of PW@CaCO_3_@8% Fe_3_O_4_ phase change microcapsules.

**Figure 3:**
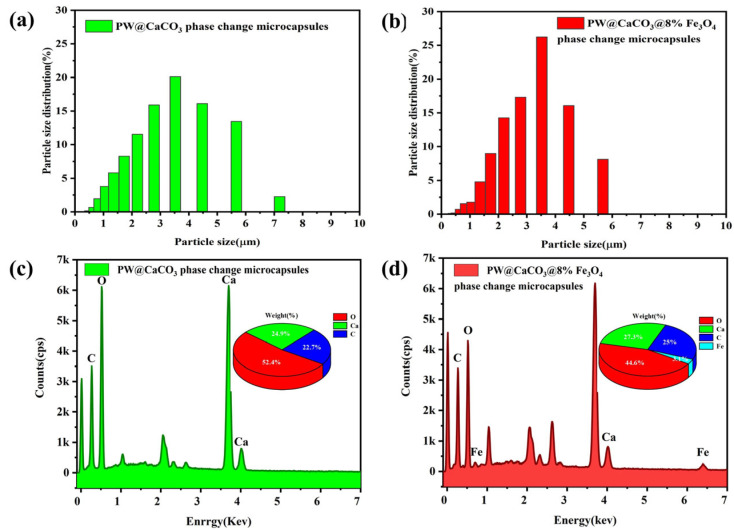
(**a**,**b**) Particle size distribution images of PW@CaCO_3_ phase change microcapsules and PW@CaCO_3_@8% Fe_3_O_4_ phase change microcapsules; (**c**,**d**) EDS image of PW@CaCO_3_ phase change microcapsules and PW@CaCO_3_@8% Fe_3_O_4_ phase change microcapsules.

The surface morphology of PW@CaCO_3_ phase change microcapsules and PW@CaCO_3_@8% Fe_3_O_4_ phase change microcapsules were characterized using scanning electron microscopy (SEM), a laser particle size (LPS) distribution analyzer, and energy spectrum mapping (EDS), as shown in Figure 2 and Figure 3. Figure 2a,b illustrates the SEM images of PW@CaCO_3_ phase change microcapsules, revealing their regular spherical shape with distinct contours and uniform dispersion. Additionally, the LPS image in Figure 3a shows that the particle size distribution range of PW@CaCO_3_ phase change microcapsules is about 2 to 6 μm. Figure 2c,d expressed the SEM images of PW@CaCO_3_@8% Fe_3_O_4_ phase change microcapsules. We observed the surface of the microcapsule sphere without obvious Fe_3_O_4_ nanoparticles. Furthermore, the LPS image in Figure 3b shows that the particle size distribution range of PW@CaCO_3_@8% Fe_3_O_4_ phase change microcapsules is about 2 to 6 μm, indicating that the addition of Fe_3_O_4_ nanoparticles did not affect the original morphology of the microcapsules. Figure 3c,d respectively, show the EDS images of PW@CaCO_3_ phase change microcapsules and PW@CaCO_3_@8% Fe_3_O_4_ phase change microcapsules. The comparison between Figure 3c,d indicates that Fe_3_O_4_ nanoparticles were successfully incorporated into the PW@CaCO_3_@8% Fe_3_O_4_ phase change microcapsules, with a Fe element content of 3.1%. Compared to the double-shell microcapsules prepared using Fe_3_O_4_ nanoparticles as a separate shell layer mentioned in some research, this doping method not only does not affect the original smoothness of the microcapsules but also the particle size of the obtained microcapsules is almost unaffected.

### 3.3. Analysis of Chemical Structure, Thermal, and Magnetic Properties of Microcapsules

**Figure 4:**
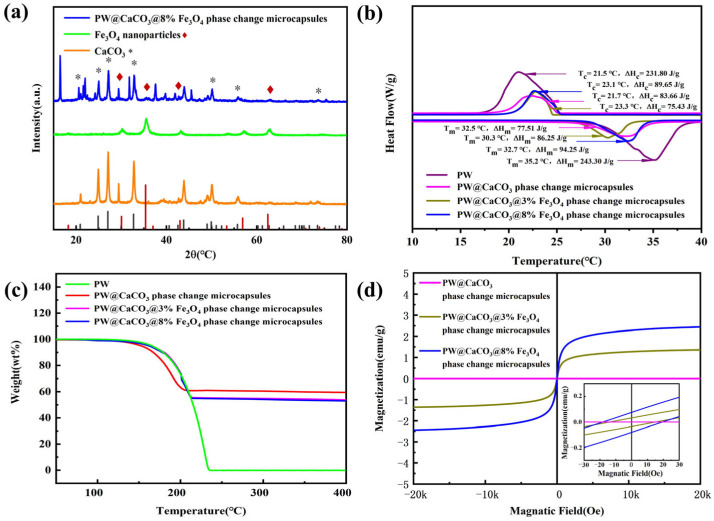
(**a**) XRD patterns of CaCO_3_, Fe_3_O_4_ nanoparticles, and PW@CaCO_3_@8% Fe_3_O_4_ phase change microcapsules; (**b**) DSC curves of PW and microcapsules; (**c**) TG curves of PW and microcapsules; (**d**) VSM curves of magnetic phase change microcapsules.

The crystal structure of CaCO_3_, Fe_3_O_4_, and magnetic phase change microcapsules is illustrated in Figure 4a. It can be observed that the diffraction peaks of magnetic PW@CaCO_3_ phase change microcapsules at 2θ angles of 20.91°, 24.87°, 27.03°, 32.71°, 49.92°, 55.73°, and 73.45°represent the characteristic diffraction peaks of CaCO_3_, corresponding to crystal faces (002), (100), (101), (102), (104), (202), and (212), respectively [16]. Additionally, 2θ angle at 30.35°, 35.39°, 43.01°, and 62.46° represent the characteristic diffraction peaks of Fe_3_O_4_ nanoparticles, which correspond to the crystal faces (220), (311), (400), and (440), respectively [17]. Thus, the curve of magnetic PW@CaCO_3_ phase change microcapsules represents both the crystal faces of CaCO_3_ and Fe_3_O_4_ nanoparticles.

The phase change behavior of PW, PW@CaCO_3_ phase change microcapsules, and magnetic phase change microcapsules with different mass fractions of Fe_3_O_4_ nanoparticles is illustrated in Figure 4b. The DSC curves of the samples shown in Figure 4b are the DSC curves obtained using DSC tests performed three times for each sample, with the enthalpy values being the median values. All samples were tested under the same conditions. It includes the enthalpy of melt crystallization and the temperature range of phase change for the above samples. From the graph, the melting enthalpy of pure PW is 243.30 J/g. The enthalpy of melting of PW@CaCO_3_ phase change microcapsules is 77.51 J/g, while the enthalpy of phase change microcapsules increased with the addition of Fe_3_O_4_ nanoparticles. Specifically, when the mass fraction of Fe_3_O_4_ nanoparticles is 8%, the enthalpy of melting reaches 94.25 J/g, demonstrating that the addition of Fe_3_O_4_ nanoparticles has a promoting effect on the thermal storage behavior of microcapsules. After the addition of Fe_3_O_4_ nanoparticles, the phenomenon of enhanced thermal storage capacity is similar to the results of Liu et al. [18], who also observed an improvement in the enthalpy value of the microcapsules after the addition of nanoparticles. This may be attributed to the increased thermal conductivity of the microcapsules.

The thermal degradation behaviors of PW, PW@CaCO_3_ phase change microcapsules, and magnetic PW@CaCO_3_ phase change microcapsules are illustrated in Figure 4c. As depicted in the TG curves, the initial decomposition temperature of PW is approximately 120 °C, and the maximum mass loss occurs around 230 °C. Similarly, the starting decomposition temperature of both PW@CaCO_3_ and magnetic PW@CaCO_3_ phase change microcapsules is also 120 °C. However, the PW@CaCO_3_@3% Fe_3_O_4_ phase change microcapsules exhibit a mass loss rate of 58% at 210 °C, the PW@CaCO_3_@8% Fe_3_O_4_ phase change microcapsules show a mass loss of 62% at 210 °C, and the PW@CaCO_3_ phase change microcapsules exhibit a mass loss of 55% at 210 °C. These results indicate that the addition of Fe_3_O_4_ nanoparticles marginally enhances the thermal stability of the microcapsules. As the temperature reached 400 °C, no further weight changes were observed, suggesting that the core material of PW had fully evaporated by this stage, leaving only Fe_3_O_4_ nanoparticles and the CaCO_3_ shell material [19].

The magnetic properties of PW@CaCO_3_ phase change microcapsules with different mass fractions of Fe_3_O_4_ nanoparticles are presented in Figure 4d. As shown in the figure, the hysteresis loops of the samples all exhibit a typical “S” shape, indicating smaller coercivity and residual magnetization strength under the applied magnetic field. Specifically, when the mass fraction of Fe_3_O_4_ nanoparticles is 3%, the magnetization saturation strength of the magnetic phase change microcapsule is 1.35 emu/g. The saturation magnetization value increases to 2.44 emu/g when the mass fraction of Fe_3_O_4_ nanoparticles is 8%. With the increase in the mass fraction of Fe_3_O_4_ nanoparticles, the saturation magnetization of the microcapsules shows an upward trend, which is consistent with the findings of Liu et al. [20].

### 3.4. Physical and Surface Morphology Analysis of Seaweed Fibers

**Figure 5:**
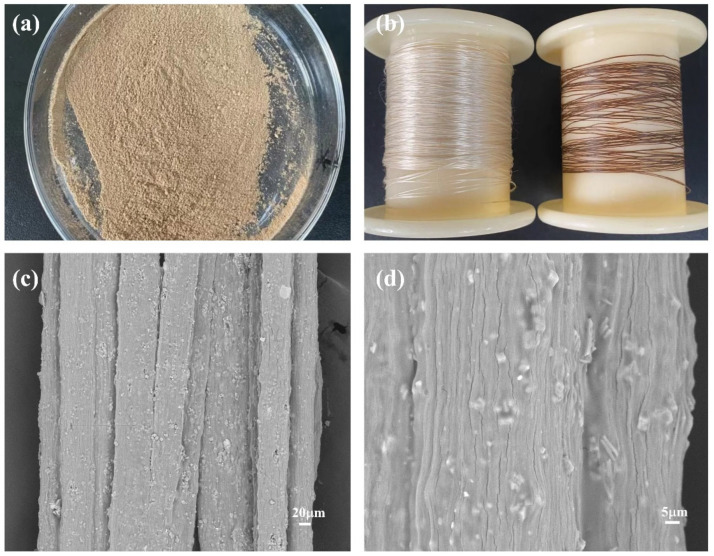
(**a**,**b**) Physical drawings of PW@CaCO_3_@8% Fe_3_O_4_ phase change microcapsules and seaweed fibers; (**c**,**d**) SEM of seaweed fibers with PW@CaCO_3_@8% Fe_3_O_4_ phase change microcapsules at different magnifications.

The physical and surface morphology of the seaweed fibers is depicted in Figure 5. In Figure 5a, the physical image of PW@CaCO_3_@8% Fe_3_O_4_ phase change microcapsules is presented. Due to the addition of brown Fe_3_O_4_ nanoparticles, the magnetic phase change microcapsules exhibit a uniform brown color. Figure 5b displays the physical drawings of the seaweed fibers. On the left side of the figure is pure seaweed fiber, and on the right side is seaweed fiber with PW@CaCO_3_@8% Fe_3_O_4_ phase change microcapsules added. It can be seen that pure seaweed fibers exhibit a light-yellow color and good luster, while seaweed fibers containing PW@CaCO_3_@8% Fe_3_O_4_ phase change microcapsules appear brown with deteriorated luster. The surface morphology of the seaweed fibers containing PW@CaCO_3_@8% Fe_3_O_4_ phase change microcapsules is shown in Figure 5c,d. It can be observed that the magnetic phase change microcapsules are distributed on the interior and surface of the seaweed fibers. Although some of the microcapsules are not completely encapsulated, the overall dispersion is good.

### 3.5. Analysis of Thermal Energy Storage Properties of Thermoregulated Seaweed Fibers

**Figure 6:**
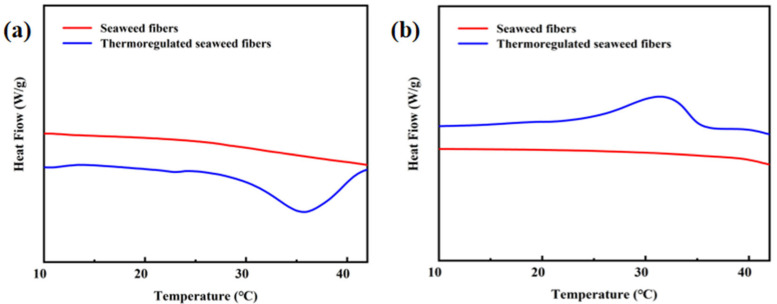
(**a**) Heating and (**b**) cooling DSC curves in seaweed fibers.

Figure 6a,b depicts the DSC curves of the seaweed fibers, while Table 1 provides detailed information on the enthalpies and temperature range. The DSC curves of the samples shown in Figure 6a,b are the DSC curves obtained using DSC tests performed three times for each sample, with the enthalpy values being the median values. All samples were tested under the same conditions. It is evident that pure seaweed fibers do not possess temperature regulation properties. The melting enthalpy and crystallization enthalpy were measured at 19.8 J/g and 19.55 J/g, respectively, with a phase transition temperature ranging from 15 to 39 °C.

**Figure 7:**
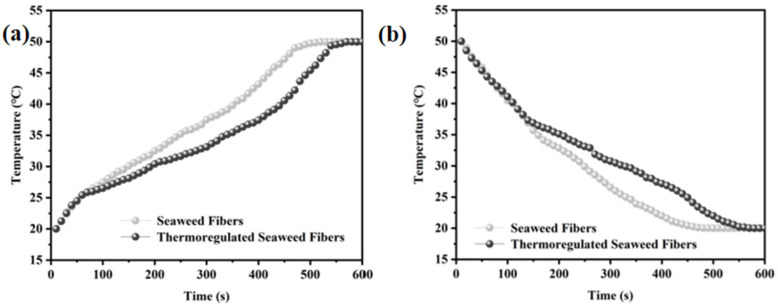
(**a**) Heating curves and (**b**) cooling curves in time-temperature graph of seaweed fibers.

Figure 7a,b shows the time-temperature curve of the sample. The specific operation is as follows: using a flat plate insulation device for heating and cooling operations, while using an infrared thermal imager to measure the temperature at intervals of 0.5 min, and finally plotting the time-temperature curve. Figure 7a illustrates the time-temperature curve of the fibers during warming [21] The seaweed fibers were simultaneously warmed from room temperature to 50 °C. Initially, both fibers exhibited a similar heating rate, increasing to over 25 °C within 1 min. Subsequently, the heating rate of the thermoregulated seaweed fibers slowed down due to the arrival of their phase transition temperature. After 9 min, both seaweed fibers reached approximately 50 °C, indicating that the phase transition properties of the thermoregulated seaweed fibers could be maintained for about 8 min during the heating process. Figure 7b presents the time-temperature curve of the fibers during cooling down [22]. The seaweed fibers were cooled from 50 °C to about 20 °C simultaneously. In the first 2 min, the obtained fibers cooled at a similar rate. Especially by the second minute, their temperatures had dropped below 37 °C. Subsequently, the cooling rate of thermoregulated seaweed fibers decreased gradually. By 9 min, the temperature of the seaweed fibers had reached about 20 °C. This indicates that thermoregulated seaweed fiber has a certain heat buffer performance, which can be maintained for 7 min during the cooling process.

## 4. Conclusions

(1) Summary of Findings: Magnetic PW@CaCO_3_ phase change microcapsules were successfully synthesized using a self-assembly method, integrating PW as the phase change core and a hybrid shell composed of CaCO_3_ and Fe_3_O_4_ nanoparticles. Characterization revealed that with an 8% mass fraction of Fe_3_O_4_ nanoparticles, the microcapsules exhibited a saturation magnetization of 2.44 emu/g and a thermal enthalpy of 94.25 J/g, highlighting their effective magnetic and thermal storage properties. Thermoregulated seaweed fibers, incorporating SA, PVA, and the magnetic phase change microcapsules, were fabricated through wet spinning. These fibers demonstrated a melting enthalpy of 19.8 J/g, significantly higher than pure seaweed fibers, with phase change behavior sustained for up to 8 min during heating.
(2) Limitations: Despite these advancements, several limitations were identified. The primary limitation is the potential variability in the thermal performance of the fibers due to inconsistencies in the dispersion of Fe_3_O_4_ nanoparticles. Additionally, the scalability of the wet spinning process for large-scale production remains a challenge. The long-term stability of the microcapsules under repeated thermal cycles has not been thoroughly tested.
(3) Future Work: Future research should focus on optimizing the dispersion of nanoparticles to enhance uniformity and performance. Investigating alternative methods for large-scale production and evaluating the long-term durability of the phase change materials under extended thermal cycling are also recommended. Additionally, exploring the integration of these materials into diverse textile applications could further demonstrate their practical benefits.

## Figures and Tables

**Table 1:** DSC data of seaweed fibers.

Samples	Melting Process		Crystallization Process	
	Tm (°C)	ΔHm (J/g)	Tc (°C)	ΔHc (J/g)
Seaweed fibers	/	0	/	0
Thermoregulated seaweed fibers	30.5	19.8	26.0	19.55

## Data Availability

The raw data supporting the conclusions of this article will be made available by the authors on request.

## References

[1] Luo L., Luo W., Chen W., Hu X., Ma Y., Xiao S., Li Q., Jiang X. (2023). Form-stable phase change materials based on graphene-doped PVA aerogel achieving effective solar energy photothermal conversion and storage. Sol. Energy.

[2] u Mekrisuh K., Giri S., Udayraj, Singh D., Rakshit D. (2021). Optimal design of the phase change material based thermal energy storage systems: Efficacy of fins and/or nanoparticles for performance enhancement. J. Energy Storage.

[3] Liu X., Yang F., Li M., Sun C., Wu Y. (2022). Development of cost-effective PCM-carbon foam composites for thermal energy storage. Energy Rep..

[4] Chen H., Xuan J., Deng Q., Gao Y. (2022). WOOD/PCM composite with enhanced energy storage density and anisotropic thermal conductivity. Prog. Nat. Sci. Mater. Int..

[5] Reyes G., Ajdary R., Yazdani M.R., Rojas O.J. (2022). Hollow filaments synthesized by dry-jet wet spinning of cellulose nanofibrils: Structural properties and thermoregulation with phase-change infills. ACS Appl. Polym. Mater..

[6] Kumar D.M., Chiranjib B., Sumit B., Murari K.P. (2022). Property-enhanced paraffin-based composite phase change material for thermal energy storage: A review. Environ. Sci. Pollut. Res..

[7] Aulakh J.S., Joshi D.P. (2022). Thermal and morphological study of paraffin/SEBS/expanded graphite composite phase change material for thermal energy storage. Energy Sources Part A Recovery Util. Environ. Eff..

[8] Chen S., Zheng Z., Liu H., Wang X. (2023). Highly efficient, antibacterial, and salt-resistant strategy based on carbon black/chitosan-decorated phase-change microcapsules for solar-powered seawater desalination. ACS Appl. Mater. Interfaces.

[9] Zhao L., Wu X., Cao Z., Hu X., Zheng S. (2023). Enhanced thermal conductivity of phase change microcapsules based on boron nitride/graphene oxide composite sheets. ACS Appl. Polym. Mater..

[10] Zhang H., Wang X., Wu D. (2010). Silica encapsulation of n-octadecane via sol-gel process: A novel microencapsulated phase-change material with enhanced thermal conductivity and performance. J. Colloid Interface Sci..

[11] Maiti T.K., Dixit P., Suhag A., Bhushan S., Yadav A., Talapatra N., Chattopadhyay S. (2023). Advancements in organic and inorganic shell materials for the preparation of microencapsulated phase change materials for thermal energy storage applications. RSC Sustain..

[12] Liu H., Wang X., Wu D. (2019). Innovative design of microencapsulated phase change materials for thermal energy storage and versatile applications: A review. Sustain. Energy Fuels.

[13] Wei H., He F., Li Y., Zhang Q., Zhou Y., Yan H., He R., Fan J., Yang W. (2019). Bifunctional paraffin@ CaCO_3_: Ce^3+^ phase change microcapsules for thermal energy storage and photoluminescence. ACS Sustain. Chem. Eng..

[14] Jiang Z., Yang W., He F., Xie C., Fan J., Wu J., Zhang K. (2018). Modified phase change microcapsules with calcium carbonate and graphene oxide shells for enhanced energy storage and leakage prevention. ACS Sustain. Chem. Eng..

[15] Ke W.D.K., Wu X.W., Zhang J.L. (2021). In situ polymerization of organic and inorganic phase change microcapsule and enhancement of infrared stealth via nano iron. Colloids Surf. A Physicochem. Eng. Asp..

[16] Liu X., Wang J., Xie H., Guo Z. (2022). Concurrent magnetic and thermal energy storage using a novel phase-change microencapsulated -nanoparticles fluid. J. Energy Storage.

[17] Lu B., Zhang Y., Zhang J., Zhu J., Zhao H., Wang Z. (2022). Preparation, optimization and thermal characterization of paraffin/nano-Fe_3_O_4_ composite phase change material for solar thermal energy storage. J. Energy Storage.

[18] Liu X., Wang J., Xie H., Guo Z. (2023). Self-assembled synthesis of microencapsulated paraffin wax phase change materials with excellent thermal properties of calcium carbonate shell. J. Enhanc. Heat Transf..

[19] Tian D., Shi T., Wang X., Liu H., Wang X. (2022). Magnetic field-assisted acceleration of energy storage based on microencapsulation of phase change material with CaCO_3_/Fe_3_O_4_ composite shell. J. Energy Storage.

[20] Liu H., Tian X., Ouyang M., Wang X., Wu D., Wang X. (2021). Microencapsulating n-docosane phase change material into CaCO_3_/Fe_3_O_4_ composites for high-efficient utilization of solar photothermal energy. Renew. Energy.

[21] Zhang M., Shen H., Qian Z., Liu H., Tian D., Wang X. (2022). Dual-purpose applications of magnetic phase-change microcapsules with crystalline-phase-tunable CaCO_3_ shell for waste heat recovery and heavy metal ion removal. J. Energy Storage.

[22] Wang X., Lei W., Zou F., Zhang C., Zhu J. (2022). Synthesis and characterization of nano-Fe_3_O_4_ hybrid phase change microcapsules for infrared stealth and microwave absorption. J. Mater. Res..

